# ACE-I/ARB Therapy prior to Contrast Exposure: What Should the Clinician Do?

**DOI:** 10.1155/2014/423848

**Published:** 2014-01-29

**Authors:** Robert Kalyesubula, Peace Bagasha, Mark A. Perazella

**Affiliations:** ^1^Makerere University College of Health Sciences, 7072 Kampala, Uganda; ^2^Section of Nephrology, Yale University School of Medicine, New Haven, CT 06520, USA

## Abstract

Contrast-induced nephropathy (CIN) is now one of the three leading causes of acute kidney injury in the world. A lot is known about the risk factors of CIN, yet it remains a major cause of morbidity, end stage renal disease, prolonged hospital stay, and increased costs as well as a high mortality. Many patients undergoing contrast-based radiological investigations are treated with angiotensin converting inhibitors (ACE-Is) or angiotensin receptor blockers (ARBs) for their cardiac and renal benefits and their known mortality benefits. However, controversy exists among clinicians as to whether ACE-Is and ARBs should be continued or discontinued prior to contrast media exposure. In this paper we review the current evidence on ACE-I/ARB therapy for patients undergoing procedures involving use of contrast media and provide recommendations as to whether these drugs should be continued or held prior to contrast exposure.

## 1. Introduction

The incidence of contrast-induced nephropathy (CIN) varies from 3.3% to 76% [1–3] depending on the population under study. According to the European Society of Urogenital Radiology (ESUR) guidelines, CIN is defined as an absolute increase in serum creatinine concentration ≥0.5 mg/dL or as a relative increase ≥25% above baseline within 3 days of contrast media exposure [4]. There are some well-described risk factors for CIN including advanced age, baseline kidney injury, diabetes mellitus, hypovolemia, nonsteroidal anti-inflammatory drug (NSAID) use, presence of a malignancy, amount and type of contrast media, and anemia [5, 6].

CIN carries an increased risk of mortality especially in the elderly population and those with underlying kidney disease [6, 7]. In a meta-analysis, CIN was consistently associated with an increased risk of cardiovascular events in 14 studies, end stage renal disease in 3 studies, and prolonged hospitalization in 11 studies, and 33 studies reported an increased risk of death [8].

Multiple interventions including N-acetylcysteine, utilization of lower osmolar agents, volume expansion with or without sodium bicarbonate, avoiding nephrotoxic medications, dialysis for contrast removal, felodipine, and dopamine have been used to prevent CIN [9]. ACE-I and angiotensin receptor blockers (ARBs) on the other hand are used extensively for patients with congestive cardiac failure, hypertension, proteinuric kidney disease, myocardial ischemia, and diabetic nephropathy. Controversy exists among clinicians as to whether the use of ACE-Is and ARBs should be continued or discontinued prior to contrast media exposure [10–13].

We review the current available evidence for continuing or withholding ACE-Is/ARBs for patients undergoing procedures involving use of contrast media.

## 2. Current Recommendations for Contrast Prophylaxis

Several interventions including volume expansion, sodium bicarbonate, N-acetylcysteine, use of low or isoosmolar agents, felodipine, withholding nephrotoxic agents, diuretics, and others have been used to prevent or reduce CIN. These have been extensively reviewed by Gleeson and Bulugahapitiya [14]. In a meta-analysis of 17 randomized trials, sodium bicarbonate prophylaxis reduced the incidence of CIN when compared to other preventive strategies for CIN but showed no significant difference in the requirement for renal replacement therapy (RRT) and mortality [15]. Although prophylactic hemodialysis or hemofiltration has been used to prevent CIN in one clinical trial, the majority of studies show no benefit or harm associated with this intervention [9, 16]. The most simple and cost effective method to prevent CIN is proper volume expansion with normal saline, avoiding nephrotoxic agents, and use of the lowest possible volume of contrast media.

## 3. Mechanism of Action of ACE-I/ARB and Possible Mechanisms for Benefit or Harm in CIN

ACE-Is act by inhibiting the renin-angiotensin-aldosterone system (RAAS), specifically the conversion of angiotensin-I to angiotensin-II, thereby causing vasodilatation of the efferent renal arterioles and thus decreasing the intraglomerular pressures [17]. They are thus called renoprotective because of this effect. ARBs on the other hand block the angiotensin-II receptors on the efferent arterioles and thus lead to lowering of both blood pressure and intraglomerular pressure. It is possible that ACE-Is and ARBs may offer a protective role by inhibiting afferent vasoconstriction that is caused by contrast media. The inhibition of angiotensin-II has been shown to prevent both vasoconstriction as well as generation of reactive oxygen species coupled with increased synthesis of nitric oxide, which is a potent vasodilator [18, 19].

**Figure 1:**
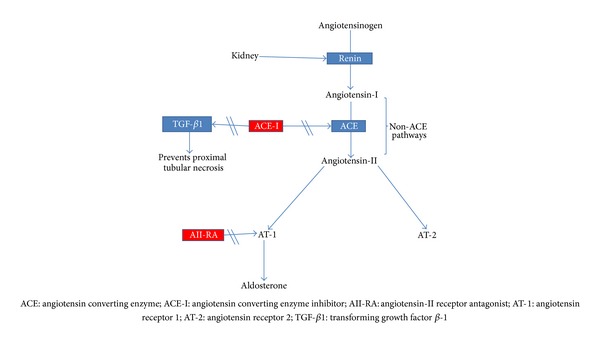
Mechanism of action of ACE-Is/ARBs. ACE-Is act to inhibit the conversion of angiotensin-I to angiotensin-II as well as the formation of transforming growth factor beta-1, which may promote proximal tubular cell injury. Angiotensin receptor antagonists (ARAs) prevent binding of angiotensin-II to its receptor. ACE-Is and ARAs major effect is to decrease intraglomerular pressures and in the setting of hypotension are associated with hypoperfusion of the kidney and a reduction in GFR.

On the other hand, ACE-Is also inhibit the formation of transforming growth factor beta-1 (TGF-*β*1) directly or through the inhibition of angiotensin-II [20]. Moreover, TGF-*β*1 has recently been shown to prevent proximal tubular cell injury and necrosis [21]. It is thus possible that ACE-Is cause their injurious effects in CIN by indirectly inhibiting formation of TGF-*β*1. The mechanisms of action of ACE-Is and ARBs are summarized in Figure 1.

## 4. Association of ACE-Is/ARBs with Increased CIN Risk

A number of researchers have found that ACE-Is exacerbate kidney failure in patients with CIN. ACE-Is decrease glomerular filtration rate through induction of systemic hypotension and the vasodilatory effect on efferent arterioles and may thus increase the risk of developing CIN. The reduction in GFR may also be further accentuated by the direct effect of the contrast, which causes afferent vasoconstriction [22].

In a randomized controlled trial, Toprak administered captopril to 48 patients and placebo to 32 patients with a baseline creatinine of less than 2 mg/dL. Five captopril treated patients (8.3%) developed CIN compared to 1 (3%) in the control group (*P* = 0.02) [23].

Hölscher et al. examined the incidence of CIN via a prospective trial in 412 patients with a baseline serum creatinine between 1.5 mg/dL and 3.5 mg/dL that required an elective left heart catheterization. They found that the use of ACE-Is as part of the preprocedural regimen was a significant independent predictor for development of CIN within 72 hours, increasing the risk more than sixfold (OR 6.16, 95% CI 2.01 to 18.93). They recommended discontinuation of ACE-Is before administration of contrast media [24].

Cirit and colleagues conducted a prospective trial to study the incidence of CIN in patients of at least 65 years of age with mild to moderate elevations of creatinine requiring nonemergent coronary angiography. They recruited 230 patients who were divided into two groups according to prior use of ACE-Is (ACE-inhibitor group, *n* = 109; control group, *n* = 121). CIN was defined as an increase in serum creatinine of at least 25% above the baseline value within 48 hours after the percutaneous coronary intervention. Patients were included in the ACE-I group if they received the drug within 2 months of the procedure. The incidence of CIN in the ACE-I group was 15.6% compared to 5.8% in the non-ACE-I group (*P* = 0.015). Chronic ACE-I administration was noted to be a risk indicator of CIN (odds ratio 3.37; 95% confidence interval 1.14–9.94; *P* = 0.028). This study concluded that in elderly patients with mild to moderate renal insufficiency, chronic use of ACE-Is before contrast increased the risk of CIN by more than 3-fold [25].

Umruddin et al. conducted a retrospective case control study to assess the influence of ACE-I or ARB use on the incidence of CIN in 201 patients undergoing coronary angiography. CIN was defined as an increase in serum creatinine >25% above baseline within 48 hours of radiocontrast exposure. The CIN group had 96 patients, and the control group had 105 patients. The 2 groups were matched for variables such as age, sex, weight, baseline serum creatinine, diabetes mellitus, dye load, use of diuretics and statins, and preprocedure prophylactic measures for CIN. They found an incidence of CIN of 4.55%. In the CIN group, 56 patients (58.3%) were on either an ACE-I or ARB while 36 (34.5%) control patients were not on these drugs (*P* < 0.001). A greater than 2-fold risk (95% confidence interval, 1.51–4.76) of developing CIN was noted with ACE-I or ARB use. The authors concluded that use of ACE-Is or ARBs is an independent risk factor for developing CIN. They recommended discontinuation of ACE-Is or ARBs at least 48 hours prior to contrast agent exposure, especially in patients with multiple risk factors [26].

In a retrospective study from Korea, 5300 patients who underwent coronary angiography and had pre- and postprocedure serum creatinine measurements were examined. 1322 patients treated with ACE-Is or ARBs and 1322 nonusers were well matched on multiple variables using propensity scoring. CIN was defined as ≥0.3 mg/dL or ≥50% increase in serum creatinine level within 48 hours after angiography. The incidence of CIN was significantly higher in ACE-I or ARB users than in nonusers (11.4% versus 6.3%). The multivariable adjusted odds ratio for CIN was 1.4 with ACE-I or ARB use. It was concluded that the use of ACE-I or ARBs during coronary angiography increases the incidence of CIN; however, randomized clinical trials to confirm the effect of ACE-I/ARB therapy on the development of CIN were recommended [27].

Kiski and colleagues performed a post hoc analysis data from 412 (83.5% men, 29.1% diabetes mellitus, and 74.6% hypertension) patients studied in the Dialysis-versus-Diuresis trial. Two hundred and sixty-nine patients (65.3%) were taking ACE-Is while 33 were on ARBs. RAAS blockade was associated with a 3-fold increase in the incidence of CIN after procedure (odds ratio 3.082, 95% confidence interval, 1.234–7.698, *P* = 0.016). It was concluded that RAAS blockade prior to contrast use is an independent predictor of CIN [10].

## 5. Protective or Neutral Effects of ACE-I/ARB in CIN

Dangas and colleagues conducted a prospective cohort study involving 7,230 patients. Of these, 1,980 had CKD (estimated glomerular filtration rate <60 mL/minute/1.73 m^2^) while 5,250 did not have underlying CKD. CIN was defined as an increase of at least 25% (or ≥0.5 mg/dL) in preprocedural serum creatinine at 48 hours after the procedure. Thirty-one percent of the CKD patients treated with an ACE-I before catheterization developed CIN compared to 32.9% who did not (*P* = 0.02). Of the non-CKD patients treated with an ACE-I before catheterization, 28.4% developed CIN compared with 24.5% who did not receive an ACE-I (*P* = 0.01). In a multivariate regression model that included preprocedural medications, taking an ACE-I was associated with a lower risk of CIN in patients with underlying CKD [7].

Rosenstock and colleagues undertook a prospective randomized controlled study in stages 3-4 CKD patients who were receiving ACE-Is or ARBs for at least 1 month before angiography. Patients were randomly assigned to discontinuation of (*n* = 113) or continuation of these drugs (*n* = 107). However, the dose of ACE-Is or ARBs in the continuation group was held on the morning of the procedure and for 24 hours afterward, confounding the results. A control group of patients who were ACE-I- or ARB-naive (*n* = 61) were included. No difference in postprocedure serum creatinine, estimated glomerular filtration rate, and incident CIN was noted in the three groups. The authors concluded that stages 3-4 CKD patients do not need to hold their ACE-Is and ARBs prior to contrast exposure [28].

Dadpey and colleagues conducted a randomized clinical trial in 60 patients assigned to various groups. Groups A and B were treated with ACE-Is and groups C and D were treated with diuretics. In group A, ACE-Is were discontinued 36 hours before percutaneous intervention (PCI) and in group C diuretics were also discontinued. The after intervention and overall increase in serum creatinine concentration were compared by ANOVA between groups. No significant increase in the serum creatinine concentrations was noted between groups A and B (0.07 ± 0.22 compared to 0.06 ± 0.13 mg/dL, resp., *P* = 0.7). Similarly, this difference was not significant between groups C and D (0.08 ± 0.17 compared to 0.05 ± 0.14 mg/dL, resp., *P* = 0.2). It was concluded that ACE-Is and diuretics have no major adverse renal effects in patients with normal kidney function undergoing PCI [29].

Gupta et al. in a randomized study among 71 diabetes mellitus patients undergoing coronary angiography found a protective effect of captopril therapy. Patients were randomly assigned to captopril 25 mg thrice daily for three days starting one hour before angiography while the control group underwent the procedure without captopril. CIN defined as a rise of 0.5 mg of serum creatinine developed in 29% of the control group, while captopril reduced the risk of developing CIN by 79%. Patients in the control group had a decline in GFR of 9.6 mL/min while those on captopril had an increase of 13 mL/min as measured by TcDTPA renal scan. The authors concluded that captopril protects against the development of CIN [30].

Spatz and colleagues retrospectively examined the incidence of CIN in 178 patients with stage 3 or 4 CKD who underwent coronary angiography. Of these, 62 patients (35%) were on ACE-Is, 12 patients (7%) were on ARBs, and 1 patient (1%) was on ACE-I/ARB combination. CIN was defined as a 25% increase of serum creatinine from baseline or an increase in serum creatinine by 0.5 mg/dL from baseline. The odds ratio for AKI on day 5 was 0.73 (95% CI, 0.31 to 1.69) for ACE-Is and 0.46 (95% CI, 0.06 to 3.70) for ARBs. On multivariate analysis, these findings remained independent of demographic variables, comorbidities, type of contrast medium, and the prophylactic strategies utilized. It was concluded that patients on RAAS blockade therapy before contrast exposure did not have an increased incidence of CIN [11].

A meta-analysis identified 7 randomized controlled trials that enrolled 792 patients undergoing intravascular angiography. The overall pooled odds ratio for development of CIN using a fixed-effects model was 0.62 (95% CI, 0.37 to 1.03, *P* = 0.06), suggesting a trend towards a reduction in CIN with ACE inhibitors. The overall pooled odds ratio for development of CIN in the diabetes mellitus subgroup using a random effects model was 0.21 (95% CI, 0.06 to 0.71, *P* = 0.01), suggesting a significant reduction in CIN with ACE-Is. In this meta-analysis, they did not find clear evidence of overall benefit associated with ACE-I therapy to prevent CIN. However, patients treated with ACE-Is had a lower mean serum creatinine and a trend toward a reduction in CIN compared with control patients (odds ratio, 0.62; 95% CI, 0.37 to 1.03) [31]. Most of the trials reviewed do not focus on hard clinical endpoints, which may carry more meaningful clinical significance such as the need for acute or chronic dialysis or mortality.

Newer biomarkers such as NGAL (Neutrophil Gelatinase-Associated Lipocalin), KIM-18 (Kidney Injury Molecule-18), and cystatin C may provide expanded insight into ACE-I/ARB-related risk for CIN, but these novel biomarkers of kidney injury are not routinely used in clinical practice to assess kidney injury or modify interventions [32–35].  Table 1 shows a summary of the studies reviewed.

## 6. Recommendation

The varied results and conclusions from available studies may be in part explained by significant differences in study populations, methodology, interventions, and the variability in CIN definitions employed among the different studies. Also, consideration and adjustment for potential risk factors contributing to ACE-I effect in CIN prevention such as baseline creatinine concentrations, volume of contrast media employed, age, diabetes mellitus, drug dose, and the type of ACEIs/ARBs varied across the studies. The Mehran score, which groups CIN risk factors for those undergoing angiography, was not used consistently across the studies making the studies more difficult to compare [36]. Use of this score would further have stratified patients and eliminated some of the inherent bias in cohorts and clinical trials for CIN studies. Notwithstanding this, the studies that show a benefit in hemodynamic parameters and GFR are limited in number. Though consistent with the known vasodilatory effects of ACE-Is/ARBs on the systemic and renal circulation, they do not address the issue of response to the hypotensive insult, which may complicate therapy with ACE-I/ARBs. Then, we have conflicting evidence from 2 larger observational studies, both of which concerned selection bias, with one suggesting the possibility of harm. The study by Dangas notes a CIN incidence of 31 versus 32.9% in patients on ACE-Is versus patients not on ACE-Is. Although statistically significant, these numbers are certainly not clinically relevant. On the other hand, in the study by, Hölscher et al., there was a clear 6-fold increase in CIN. Based on these data, we believe that it is not appropriate to pursue unknown benefit at the risk of potential harm. Given the lack of convincing renal benefit associated with continuing ACE-Is/ARB prior to angiography and the real possibility of harm, we recommend holding these drugs prior to contrast administration when the risk for CIN is significant.

From the evidence reviewed above, there is a clear need for large randomized clinical trials to break equipoise. The clinical trial by Mehta of McMaster under registration NCT00317252 will hopefully provide some meaningful information to guide the decision making regarding the approach of withholding or continuing ACE-Is or ARBs before contrast requiring procedures. It is also important that future studies provide hard endpoints such as the need for dialysis, development of ESRD, or mortality rather than just the rise in creatinine levels as part of the major outcomes.

## 7. Bottom Line

The data regarding the temporary discontinuation of ACE inhibitors in patients receiving contrast media are conflicting, whereas the data regarding ARBs are very limited. Patients who develop CIN have increased morbidity, mortality, length of hospital stay, and associated costs. Therefore, if there is a potential to prevent CIN by temporarily discontinuing ACE-Is (one day before the procedure and 3 days after administration of contrast) in high-risk patients, it would seem that the potential benefits outweigh the risks of short-term ACE-I discontinuation. Our view is that the most prudent approach is to withhold these drugs prior to contrast exposure and reinstitute therapy when kidney function has stabilized following the procedure.

## Figures and Tables

**Table 1:** Studies indicating harm or benefit of ACE-I or ARB in CIN.

Reference, year	Study population	Sample size	Study type	Brief description	Key findings and comments
Dangas et al., 2005 [7]	CKD and non-CKD	7,230	P.O	1,980 pts with CKD and 5,250 without CKD used to determine predictors of CIN in PCI	Taking an ACE-I was associated with a lower risk of CIN in CKD pts

Toprak, 2006 [23]	Near normal renal function	80	RCT	42 pts received captopril while 32 controls received no captopril	CIN occurred 2.5x more in the captopril group

Dadpey et al., 2007 [29]	Normal renal function undergoing PCI	240	RCT	60 pts in each of four gps of ACI-I, diuretics, with a 36 hours discontinuation of these drugs as controls	Neither diuretic nor ACE-I discontinuation or continuation increased the risk of CIN

Spatz et al., 2012 [11]	Stage III-IV CKD	178	R	Pts were either on ACE-I, ARB, or both	RAAS blockade before PCI did not increase CIN risk

Bariş et al., 2013 [37]	Near normal renal function	295	P.O	Pts in 3gps of ACE-I (*n* = 106) ARB (*n* = 94), and control group on no RAAS (*n* = 95)	Chronic usage of ACE-I and ARB increases the risk of CIN

Cirit et al., 2006 [25]	>65 yrs with mild-moderate CKD	230	P.O	One gp on ACE-I for 2 months versus gp without ACE-I before PCI	ACE-I increased risk of CIN

Rosenstock et al., 2008 [28]	Stage III-IV CKD	281	RCT	Pts on ACE-I/ARBs with therapy stopped 24 hrs before or continued compared to control before PCI	No difference in postprocedure creatinine, eGFR, and incident CIN in the 3 groups

Hölscher et al., 2008 [24]	Adults with creatinine of 1.5 to 3.5 mg/dL	412	Post hoc RCT	ACE-I administered to pts for elective PCI in the Dialysis-versus-Diuresis (DVD) trial	ACE-I therapy increased risk of CIN 6-fold

Umruddin et al., 2012 [26]	Pts with multiple risk factors	201	R, case control	Exposure to ACE-I/ARB was determined in the two gps	Exposure to ACE-I or ARB doubled the risk of CIN

Rim et al., 2012 [27]	Adults undergoing elective PCI.	5,300	R	Study compared 1322 users of ACE-I or ARBs and 1322 nonusers matched by propensity scoring	CIN was higher in ACE-I/ARB than in nonusers (11.4% versus 6.3%; *P* < 0.001)

Gupta et al., 1999 [30]	Adults with diabetes mellitus	71	RCT	Captopril administered 1 hr before angiography versus none for the control gp	Exposure to captopril reduced risk of CIN by 79%

Li et al., 2012 [31]	Pts in RCTs involving ACE-Is	792	Meta-analysis of RCTS	Searches in PubMed, MEDLINE, the Cochrane Central Register of Controlled Trials, and ISI Web of Science for impact of the ACE-Is on frequency of CIN	[ACE-Is use protective] in pts with diabetes mellitus but showed no protection or harm in other pts

Abbreviations: ACE-I: angiotensin converting enzyme inhibitor; ARBs: angiotensin receptor blockers; CIN: contrast-induced nephropathy; CKD: chronic kidney disease; e-GFR: estimated glomerular filtration rate; Gp: group; PCI: percutaneous coronary intervention; Pts: patients; P.O: Prospective observational; RAAS: renin angiotensin aldosterone system; R: retrospective; RCT: randomized controlled trial.
